# PVN mechanisms in OSA comorbidities: from intermittent hypoxia–stress to therapy

**DOI:** 10.3389/fneur.2026.1720898

**Published:** 2026-05-28

**Authors:** Haiying Sun, Chuan Cheng, Yun Zhu

**Affiliations:** 1 Department of Otorhinolaryngology, Union Hospital, Tongji Medical College, Huazhong University of Science and Technology, Wuhan, China; 2 Information and Data Center, Union Hospital, Tongji Medical College, Huazhong University of Science and Technology, Wuhan, Hubei, China

**Keywords:** chronic intermittent hypoxia, chronic stress, depression, hypertension, obstructive sleep apnea, paraventricular nucleus of the hypothalamus

## Abstract

Obstructive Sleep Apnea (OSA) is a common sleep-disordered breathing condition characterized by recurrent upper airway collapse, chronic intermittent hypoxia (CIH), and sleep fragmentation. Beyond daytime dysfunction, OSA is strongly associated with cardiovascular and neuropsychiatric comorbidities, particularly hypertension and depression. The paraventricular nucleus of the hypothalamus (PVN), a central hub for autonomic regulation and stress integration, plays a pivotal role in mediating these outcomes. This review synthesizes recent evidence on the synergistic interplay between CIH and chronic stress within the PVN. We highlight three core mechanisms—neuronal plasticity, neuroinflammation and oxidative stress, and epigenetic reprogramming—that collectively drive sustained sympathetic overactivation and hypothalamic–pituitary–adrenal (HPA) axis dysregulation. These central alterations form the neurobiological basis of OSA-related hypertension and contribute to shared pathways with mood disorders, including oxidative stress and ferroptosis. Finally, we summarize emerging diagnostic and therapeutic advances, such as non-invasive biomarkers, phenotype-specific pharmacotherapies, and precision neuromodulation approaches. Future directions include the development of composite animal models, targeted epigenetic interventions, and circuit-specific modulation strategies. Together, these insights provide a framework for mechanism-based and stratified management of OSA and its comorbidities.

## Introduction

### The pathophysiological Nexus of OSA and the central–cardiovascular axis

Obstructive Sleep Apnea (OSA) is a prevalent sleep-disordered breathing disorder characterized by recurrent upper airway collapse, chronic intermittent hypoxia (CIH), and sleep fragmentation (1). Affecting up to one quarter of adult males worldwide, OSA is increasingly recognized as a systemic disease rather than a purely mechanical airway disorder (2, 3). In addition to excessive daytime sleepiness and impaired quality of life, robust clinical and epidemiological evidence has linked OSA to a wide spectrum of cardiovascular and neuropsychiatric comorbidities, including hypertension, coronary artery disease, stroke, anxiety, and depression (4–6). These comorbidities substantially contribute to morbidity and mortality in OSA patients and often persist despite adequate correction of upper airway obstruction, suggesting the involvement of central pathophysiological mechanisms (7).

### Microanatomy, physiology, and connectomics of the paraventricular nucleus

The paraventricular nucleus of the hypothalamus (PVN) occupies a central position in the integration of autonomic, neuroendocrine, and stress-responsive functions. Classical neuroanatomical studies established the PVN as a highly heterogeneous structure composed of distinct neuronal subtypes that differ in morphology, neurochemical identity, projection targets, and physiological roles. Broadly, PVN neurons are categorized into magnocellular and parvocellular populations, a distinction that remains fundamental for understanding PVN function and dysfunction.

Magnocellular neurons, primarily located in the lateral and posterior PVN, synthesize and release the neuropeptides oxytocin and vasopressin. These neurons project predominantly to the posterior pituitary, where they regulate systemic processes such as fluid homeostasis, parturition, lactation, and social-affective behaviors. While traditionally viewed through a neuroendocrine lens, accumulating evidence indicates that magnocellular PVN neurons also influence central autonomic and emotional circuits via collateral projections, challenging the notion that their actions are exclusively peripheral.

In contrast, parvocellular PVN neurons exhibit greater functional and anatomical diversity. Neuroendocrine parvocellular neurons expressing corticotropin-releasing hormone (CRH) and thyrotropin-releasing hormone (TRH) project to the median eminence and regulate the hypothalamic–pituitary–adrenal (HPA) and hypothalamic–pituitary–thyroid axes, respectively. These neurons are subject to extensive top-down modulation from limbic and cortical regions, rendering them particularly sensitive to chronic stress and affective states. Dysregulation of parvocellular CRH neurons represents a core mechanism underlying sustained HPA axis activation in stress-related disorders.

A second major class of parvocellular neurons comprises presympathetic neurons, which project directly or indirectly to autonomic control centers, including the rostral ventrolateral medulla and the intermediolateral cell column of the spinal cord. These neurons provide a crucial anatomical substrate linking PVN activity to sympathetic outflow and cardiovascular regulation. Importantly, presympathetic PVN neurons are embedded within dense local microcircuits, receiving excitatory glutamatergic input, inhibitory GABAergic control, and neuromodulatory signals from both central and peripheral sources.

From a connectomic perspective, the PVN functions not as an isolated nucleus but as a hub within a distributed autonomic–neuroendocrine network. It integrates ascending interoceptive and viscerosensory signals from the nucleus tractus solitarius and circumventricular organs with descending cognitive–emotional inputs from the prefrontal cortex, hippocampus, and amygdala. This convergence of bottom-up and top-down information enables the PVN to coordinate adaptive responses to metabolic, cardiovascular, and psychosocial challenges, but also renders it vulnerable to maladaptive plasticity under conditions of chronic intermittent hypoxia and stress. Importantly, this network-based view argues against over-attributing OSA-related comorbidities to the PVN alone. The nucleus tractus solitarius provides primary viscerosensory input, the rostral ventrolateral medulla serves as a major downstream effector of sympathetic outflow, and limbic structures such as the amygdala shape stress-related autonomic responses.

Critically, although this classical framework of PVN organization provides an essential foundation, it does not fully capture the dynamic and plastic nature of PVN circuits. Recent advances using cell-type–specific labeling, electrophysiology, and circuit manipulation have revealed that PVN neuronal subtypes exhibit differential susceptibility to synaptic remodeling, neuroinflammatory signaling, and epigenetic reprogramming. These insights underscore the need to interpret PVN dysfunction in OSA-related comorbidities not as a uniform process, but as the outcome of cell-type– and circuit-specific maladaptation, a theme that underpins the mechanistic sections developed in the remainder of this review.

### CNS mechanisms with a focus on the PVN

Growing evidence indicates that the central nervous system (CNS) plays a critical role in integrating hypoxic and stress-related signals in OSA. Among CNS structures, the PVN appears to function as an important integrative hub coordinating autonomic output, neuroendocrine activity, and stress adaptation within a broader distributed network., a concept that has been progressively refined through experimental work by Toney and colleagues and others using electrophysiological and circuit-based approaches (8, 9). The PVN is composed of heterogeneous neuronal populations, including corticotropin-releasing hormone (CRH) neurons, presympathetic glutamatergic neurons, oxytocinergic neurons, and local GABAergic interneurons. These neuronal subtypes are distinguished by their neurotransmitters, projection targets, and functional roles. CRH neurons primarily regulate HPA axis activity, whereas presympathetic PVN neurons project to the intermediolateral cell column of the spinal cord and brainstem autonomic nuclei to directly modulate sympathetic outflow. GABAergic interneurons provide inhibitory gating of PVN output, while oxytocinergic neurons exert sympatho-inhibitory and cardioprotective effects.

Beyond intrinsic changes within the PVN, dysregulation of CRH neurons must be considered within a broader top-down regulatory network. Converging evidence indicates that chronic stress and hypoxia-associated arousal impair inhibitory influences from the prefrontal cortex and hippocampus, regions that normally constrain CRH neuron activity via polysynapticaptically organized circuits. While intrinsic plasticity of CRH neurons contributes to sustained HPA axis activation, existing data suggest that PVN dysregulation is unlikely to originate exclusively within the nucleus itself. Rather, dysfunction emerges from an interaction between weakened cortical–limbic regulation and maladaptive intrinsic PVN responses. Importantly, the relative contribution and temporal hierarchy of these processes remain unresolved, highlighting a critical gap in current models of OSA-related stress dysregulation.

Under physiological conditions, the coordinated activity of these PVN neuronal subtypes ensures appropriate adaptation to environmental challenges. However, under the dual stressors of CIH and chronic psychological stress, this finely tuned system becomes dysregulated (8, 9). Experimental studies demonstrate that CIH and stress induce distinct yet convergent forms of functional plasticity across PVN neuronal populations. CRH neurons exhibit enhanced excitatory synaptic drive and impaired top-down inhibitory control from higher brain regions such as the prefrontal cortex and hippocampus, leading to sustained HPA axis activation. Concurrently, presympathetic neurons display increased intrinsic excitability and synaptic remodeling, resulting in persistent sympathetic overactivation. In contrast, inhibitory and protective influences mediated by GABAergic and oxytocinergic neurons are attenuated, further shifting the balance toward excitation (10, 11).

**Figure 1:**
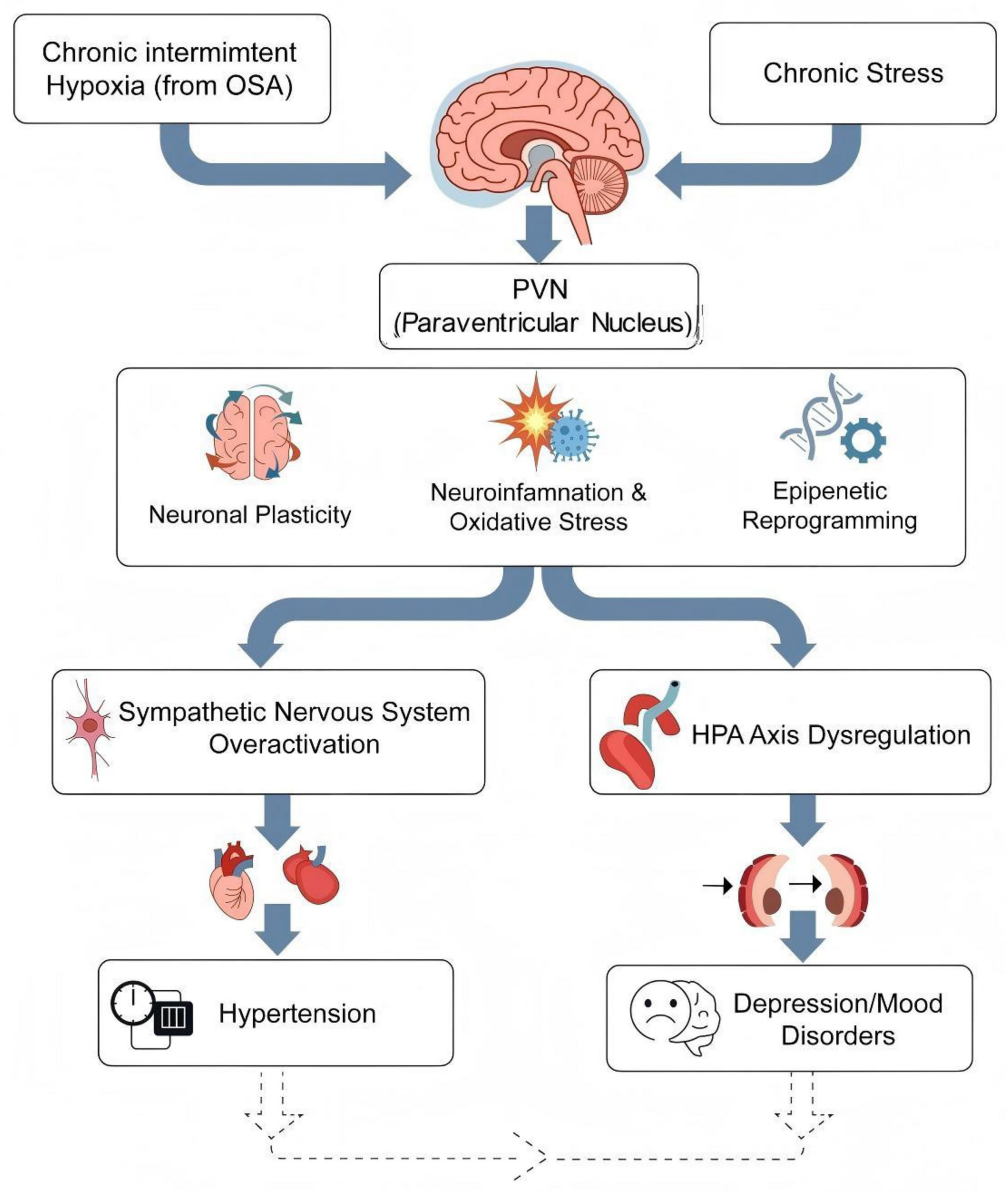
The central mechanism of synergistic action between CIH and chronic stress in the PVN and its systemic consequences. Obstructive sleep apnea (OSA) leads to chronic intermittent hypoxia (CIH), while psychosocial factors contribute to chronic stress. Both stressors converge on the paraventricular nucleus of the hypothalamus (PVN), where they synergistically promote pathological changes through three core mechanisms: (1) Neuronal plasticity, characterized by enhanced excitatory (glutamatergic) and diminished inhibitory (GABAergic) transmission; (2) Neuroinflammation and oxidative stress, involving glial cell activation, release of pro-inflammatory cytokines (e.g., IL-1β, TNF-*α*), and excessive production of reactive oxygen species (ROS); and (3) Epigenetic reprogramming, such as altered histone acetylation and DNA methylation. These central alterations lead to two primary outputs: sustained activation of the sympathetic nervous system (SNS) and dysregulation of the hypothalamic–pituitary–adrenal (HPA) axis. Ultimately, these outputs drive the development of systemic comorbidities, including hypertension (via RAAS activation and vasoconstriction) and depression/mood disorders (via mechanisms like ferroptosis and limbic circuit dysregulation), which in turn can exacerbate chronic stress, creating a vicious cycle.

Importantly, the effects of CIH and chronic stress on the PVN are not merely additive but synergistic. Hypoxia-driven oxidative stress and neuroinflammatory signaling interact with stress-induced neuroendocrine alterations, amplifying maladaptive plasticity within PVN circuits. This convergence at the PVN may offer a plausible mechanistic framework for understanding the association OSA, hypertension, and mood disorders, as well as for the limited efficacy of airway-centered therapies in fully reversing systemic complications (Figure 1).

Before detailing specific molecular and cellular mechanisms, it is important to conceptualize these alterations as components of a broader process of PVN dysregulation. Rather than reflecting isolated responses to chronic intermittent hypoxia or stress, accumulating evidence indicates that PVN dysfunction emerges from coordinated disturbances at the neuronal, neurotransmitter, and neurohumoral levels. This framework provides a unifying lens through which the diverse findings summarized below can be interpreted, and distinguishes dysregulation from transient physiological activation.

### Aims of the review

In this review, we synthesize recent experimental and clinical evidence, with an emphasis on studies published between 2023 and 2025, to elucidate how CIH and chronic stress converge on the PVN to drive OSA-related comorbidities. We focus on three interrelated mechanisms—neuronal plasticity, neuroinflammation and oxidative stress, and epigenetic reprogramming—that collectively reshape PVN function and promote sustained sympathetic and HPA axis dysregulation. Finally, we discuss emerging diagnostic and therapeutic strategies informed by these mechanistic insights, highlighting the potential of phenotype-targeted pharmacotherapy and neuromodulation to enable precision management of OSA and its systemic consequences.

## The vicious cycle: pathophysiological crosstalk between CIH and chronic stress

OSA itself constitutes a potent endogenous chronic stressor (9, 12). The recurrent nocturnal episodes of hypoxia/hypercapnia, frequent microarousals, and sleep fragmentation directly activate the hypothalamic–pituitary–adrenal (HPA) axis, leading to dysregulated cortisol secretion rhythms and elevated levels (9, 12). Clinical studies have confirmed a significant positive correlation (*r* = 0.68) between the arousal index and plasma cortisol levels in OSA patients, providing direct evidence of its role as a biological stressor (13). Furthermore, the resultant daytime sequelae, such as excessive sleepiness and cognitive impairment, create a persistent psychosocial stressor, markedly increasing the risk for anxiety and depression.

**Figure 2:**
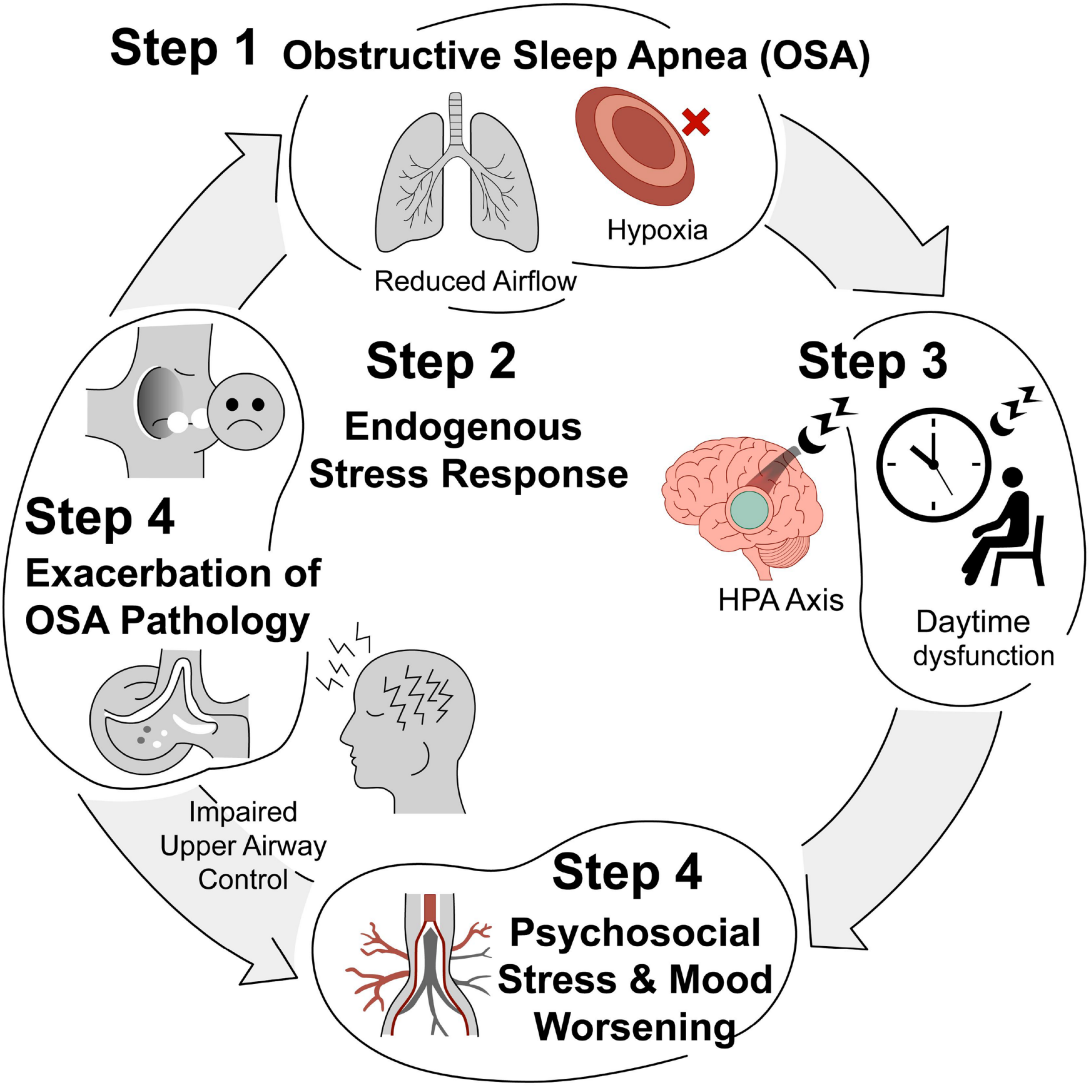
Proposed vicious cycle linking obstructive sleep apnea, endogenous stress activation, daytime dysfunction, psychosocial stress, and worsening upper airway control. Obstructive sleep apnea (OSA) is characterized by recurrent reductions in airflow and intermittent hypoxia during sleep. These events may activate endogenous stress-response pathways, including hypothalamic–pituitary–adrenal (HPA) axis signaling. Sustained stress activation can contribute to sleep fragmentation, daytime dysfunction, fatigue, and neurobehavioral impairment. Daytime symptoms and psychosocial stress may further worsen mood regulation and autonomic balance, while impaired upper airway neuromuscular control may increase vulnerability to recurrent airway obstruction. Together, these processes form a self-reinforcing cycle in which OSA-related physiological stress and psychosocial consequences interact to aggravate OSA pathology.

Conversely, exogenous chronic psychological stress can exacerbate the pathophysiology of OSA through a negative feedback loop (14, 15). A sustained state of stress activates the sympathetic nervous system, which not only directly elevates blood pressure and heart rate but may also compromise upper airway patency by impairing the neural control and tone of pharyngeal dilator muscles (16). Moreover, stress-induced chronic HPA axis hyperactivity and hypercortisolemia can centrally dysregulate respiratory control, thereby worsening the severity of nocturnal hypoxic events (17, 18). Thus, CIH and chronic stress are causally intertwined, creating a self-amplifying vicious cycle that propels disease progression (Figure 2).

## Core mechanisms: synergistic actions of CIH and chronic stress in the PVN

### PVN neuronal plasticity: remodeling the excitatory-inhibitory balance

The heightened excitability of presympathetic neurons within the PVN may represent an important upstream modulatory influence in OSA-related hypertension, acting in concert with downstream medullary sympathetic generators. Sympathetic overactivity induced by chronic intermittent hypoxia (CIH) arises from a distributed central autonomic network, in which PVN-driven excitatory inputs amplify the activity of respiratory-modulated presympathetic neurons in the rostral ventrolateral medulla (RVLM), ultimately increasing sympathetic outflow. CIH and chronic stress act in concert to disrupt the delicate synaptic homeostasis in the PVN through a “dual-hit” mechanism of enhancing excitation and diminishing inhibition (19).

Synergistic Enhancement of Excitatory Synaptic Transmission: Mechanistic studies by Wang and colleagues demonstrated that CIH potentiates PVN neuronal sensitivity to glutamate by upregulating phosphorylation of the AMPA receptor subunit GluA1 and promoting its trafficking to the postsynaptic membrane, using combined molecular and synaptic analyses in hypothalamic circuits. However, these findings are largely derived from CIH-driven hypertension models, and the extent to which similar AMPA-dependent synaptic remodeling occurs in combined CIH–stress paradigms relevant to clinical OSA remains to be determined. Concurrently, chronic stress increases the density of glutamatergic and noradrenergic terminals on the dendrites of corticotropin-releasing hormone (CRH) neurons within the PVN while weakening the “top-down” inhibitory control from the prefrontal cortex (PFC) and hippocampus. This confluence of mechanisms locks PVN neurons in a state of disinhibited hyperexcitability (20–24).

Synergistic Attenuation of Inhibitory Synaptic Transmission: CIH has been shown to downregulate the function of GABA-A receptors in the PVN, significantly impairing GABAergic inhibitory neurotransmission. This reduction in inhibitory input, coupled with enhanced excitatory drive, leads to depolarization of the resting membrane potential, an increase in input resistance, and a lower action potential threshold in PVN neurons, culminating in a marked increase in intrinsic excitability (19, 21, 25–27).

Together, these forms of synaptic and intrinsic remodeling illustrate the neuronal dimension of PVN dysregulation, characterized by persistent hyperexcitability rather than adaptive activation.

### Neuroinflammation and oxidative stress: a self-amplifying pathological storm

Glial cell-mediated neuroinflammation and oxidative stress may serve as an important pathological link between CIH and chronic stress.

Glial Activation and Pro-inflammatory Cytokine Release: Both CIH and chronic stress activate microglia and astrocytes in the PVN, triggering the release of pro-inflammatory cytokines such as interleukin-1β (IL-1β) and tumor necrosis factor-*α* (TNF-α). Mechanistic work by Wei and colleagues demonstrated that CIH activates PVN microglia via P2Y12-dependent signaling, triggering IL-1β release that directly enhances neuronal excitability and sympathetic outflow, thereby supporting the existence of a neuroimmune pathway linking CIH to autonomic dysregulation (28–31). Nevertheless, most available studies rely on region-restricted microglial manipulations, and whether distinct PVN neuronal subtypes (e.g., CRH vs. presympathetic neurons) exhibit differential vulnerability to microglial signaling remains unresolved.

The Vicious Cycle of Oxidative Stress and Inflammation: The hypoxia-reoxygenation cycles inherent to CIH are a potent source of oxidative stress, generating excessive reactive oxygen species (ROS) through the activation of NADPH oxidase (NOX) and mitochondrial damage. Chronic stress further exacerbates this process. ROS and neuroinflammation are locked in a pernicious feedback loop: ROS can activate signaling pathways like NF-κB to upregulate pro-inflammatory gene expression, while inflammatory cytokines can, in turn, promote further ROS production, creating a self-perpetuating ‘oxidative-inflammatory storm’ that may contribute to PVN neuronal dysfunction and sustained sympathetic dysregulation (32, 33).

ROS-driven neuroinflammation in intermittent hypoxia should be conceptualized not as a localized PVN event, but as part of a distributed oxidative–inflammatory storm. Ascending viscerosensory and chemoreflex signals from the nucleus tractus solitarius convey hypoxia-related information to hypothalamic circuits, while humoral and descending inputs from lamina terminalis structures integrate systemic and fluid-balance signals. Within this network, PVN microglial activation and redox signaling function as an amplification node rather than an isolated origin. This cross-level integration sustains medullary-driven sympathetic excitation and reinforces intermittent hypoxia–hypertensive responses, underscoring that PVN dysregulation emerges from bidirectional brainstem–hypothalamic interactions rather than a purely top-down process.

At this level, microglial activation and redox signaling function not as parallel phenomena, but as amplifiers of PVN dysregulation that stabilize maladaptive neuronal states.

### Epigenetic regulation: the molecular imprint of pathological memory

Epigenetic regulation may provide an important molecular interface through which chronic intermittent hypoxia and stress exert long-lasting effects on PVN function, hypoxia- and stress-related epigenetic mechanisms converge on a shared outcome—persistent transcriptional reprogramming of PVN neurons.

Under intermittent hypoxia, stabilization of hypoxia-inducible factors (HIFs) initiates transcriptional programs that extend beyond acute oxygen sensing. Emerging evidence indicates that HIF signaling interacts with chromatin-modifying enzymes, including histone deacetylases (HDACs), thereby influencing histone acetylation status and accessibility of stress- and autonomic-related gene loci. These modifications facilitate sustained expression of genes involved in excitatory neurotransmission, oxidative stress responses, and inflammatory signaling within the PVN (34, 35).

Chronic stress further reinforces this epigenetic landscape through alterations in DNA methylation and histone modification at promoters regulating corticotropin-releasing hormone and other stress-responsive genes. Importantly, these stress-induced epigenetic changes do not operate independently of hypoxia-driven mechanisms; instead, they synergize to stabilize maladaptive transcriptional states. In this context, epigenetic regulation functions less as a binary switch and more as a form of molecular memory, maintaining PVN hyperresponsiveness even after the initial hypoxic or stressor exposure has subsided (36).

Critically, while epigenetic reprogramming offers a plausible integrative framework for the persistence of PVN dysregulation, current evidence remains largely indirect and model-dependent. Most studies infer epigenetic involvement from pharmacological or correlative approaches, underscoring the need for future work employing cell-type–specific and locus-resolved epigenomic techniques to delineate causal mechanisms.

## Pathogenesis of comorbidities: from central hub to systemic cascade

### From the PVN to hypertension: sustained activation of the sympathetic-RAAS axis

Driven by the synergistic actions of chronic intermittent hypoxia and chronic stress, sustained hyperexcitability of PVN presympathetic neurons facilitates sympathetic outflow to peripheral organs, including the kidney. Increased renal sympathetic nerve activity promotes renin release and activates the renin–angiotensin–aldosterone system (RAAS), thereby contributing to the development and maintenance of neurogenic hypertension. Experimental studies in spontaneously hypertensive rats demonstrate that renal denervation during the established phase of hypertension significantly reduces blood pressure, attenuates cardiac hypertrophy, and suppresses sympathetic activity, highlighting the critical role of renal sympathetic nerves in sustaining RAAS-dependent hypertension (37). In addition to renal sympathetic mechanisms, renovascular hypertension has been shown to be mediated by angiotensin II receptors in the carotid bodies, linking RAAS activation to enhanced peripheral chemoreflex sensitivity and sympathetic drive (38). Systemically generated angiotensin II (Ang II) can then act back on the highly expressed AT1 receptors in the PVN, creating a powerful positive feedback loop that further exacerbates PVN excitability and sympathetic output. This PVN-associated vicious cycle of the ‘sympathetic-RAAS axis’ may represent an important pathophysiological process contributing to the transition of OSA-related hypertension from a neurogenic origin to a persistent, treatment-resistant state (39, 40).

### From the PVN to depression: shared pathways of oxidative stress and ferroptosis

The comorbidity rate of depression in OSA patients is as high as 35%. A recent integrative bioinformatics study provided supportive molecular evidence for this association, identifying 24 core shared genes, including superoxide dismutase 2 (SOD2) and macrophage migration inhibitory factor (MIF), and pinpointing oxidative stress, ferroptosis, and inflammatory pathways as the key biological links between the two disorders (41).

Ferroptosis is a novel form of regulated cell death that is iron-dependent and driven by lipid peroxidation, and it is intricately linked with oxidative stress and inflammation. Chronic stress has been shown to induce iron accumulation and promote ferroptosis in emotion-related brain regions like the hippocampus, leading to depressive-like behaviors. Given that CIH involves substantial oxidative stress generated by hypoxia-reoxygenation cycles, it is biologically plausible that this condition may engage pathways relevant to ferroptosis. The synergistic effects of CIH and chronic stress within the PVN and its associated emotional circuits may contribute to neuronal injury and dysfunction through oxidative stress-related pathways that could involve ferroptosis, thereby potentially increasing vulnerability to depressive symptoms (42).

Although ferroptosis is often discussed as a generic oxidative cell death pathway, its impact within the PVN is unlikely to be uniform across neuronal populations. Neuronal subtypes differ markedly in metabolic activity, iron utilization, and antioxidant capacity, suggesting that stress- and autonomic-related PVN neurons may exhibit heightened vulnerability to ferroptotic stress. In this context, ferroptosis is better conceptualized as a selective stress-amplifying mechanism that exacerbates PVN dysregulation, rather than as a deterministic fate of specific neuron classes. Importantly, direct evidence defining ferroptosis susceptibility at the level of individual PVN neuronal subtypes remains limited, highlighting a critical gap for future investigation.

## Clinical translation of core mechanisms: novel diagnostic and therapeutic strategies

### Novel biomarkers and non-invasive assessment technologies

To achieve precise and non-invasive assessment of OSA-related hypertension, technologies based on novel biomarkers such as pulse transit time (PTT) are showing great promise. PTT, measured non-invasively and continuously, is strongly and negatively correlated with blood pressure, demonstrating a diagnostic sensitivity of 85% and specificity of 88% for systolic blood pressure. Its primary advantage is the avoidance of microarousals caused by the cuff inflation of traditional ambulatory blood pressure monitoring (ABPM), making it particularly suitable for accurately assessing nocturnal blood pressure fluctuations in OSA patients (43).

### Phenotype-targeted pharmacotherapy and neuromodulation

**Figure 3:**
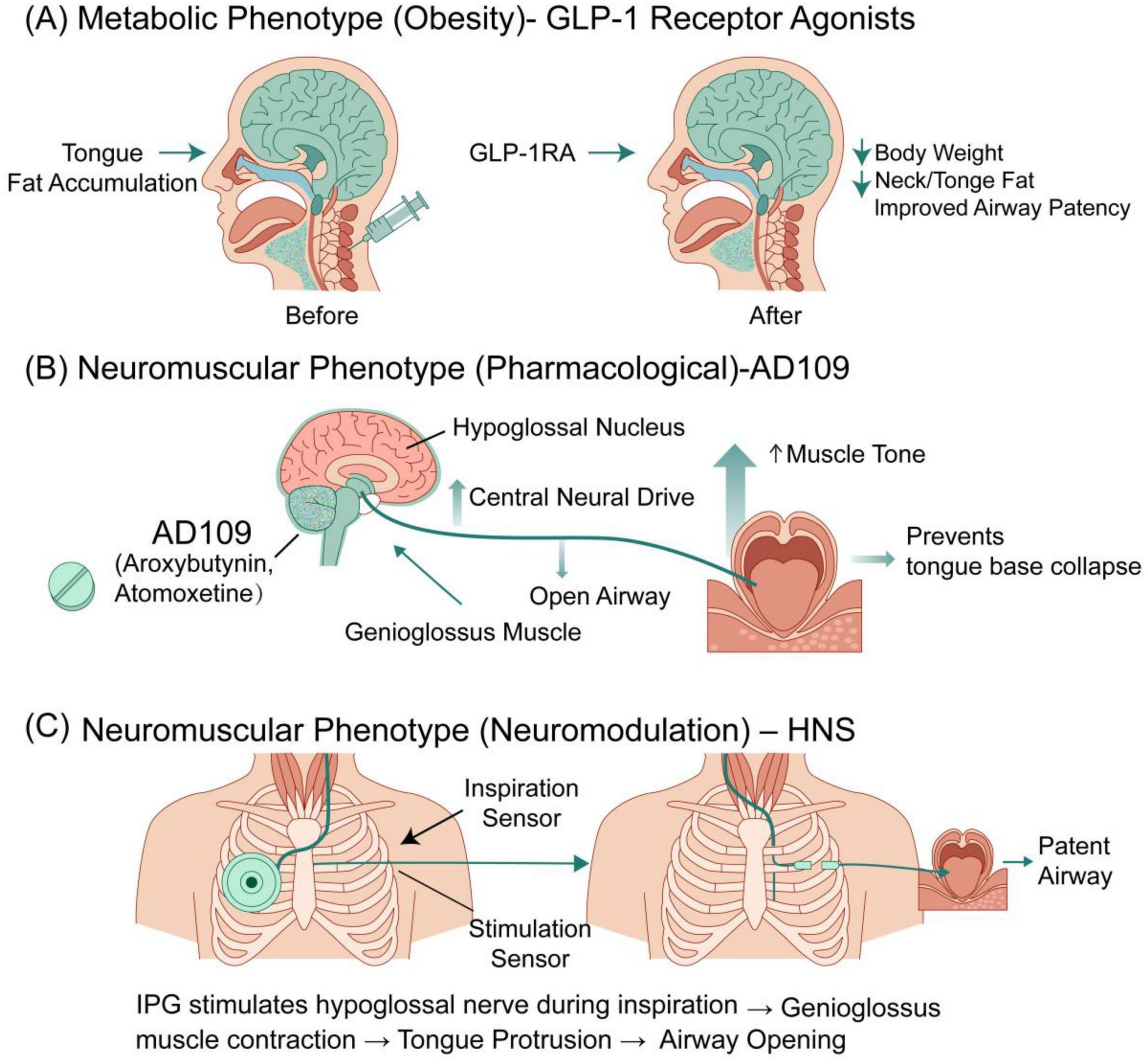
Mechanisms of action of novel phenotype-targeted therapies for obstructive sleep apnea. **(A)** Metabolic phenotype (obesity): GLP-1 receptor agonists (e.g., tirzepatide) reduce body weight and neck/tongue fat, thereby improving upper airway patency. **(B)** Neuromuscular phenotype (pharmacological): AD109 enhances central neural drive to the hypoglossal nucleus, increases genioglossus muscle tone, and prevents tongue-base collapse. **(C)** Neuromuscular phenotype (neuromodulation): Hypoglossal nerve stimulation (HNS) synchronizes with inspiration to activate the genioglossus muscle, resulting in tongue protrusion and maintenance of a patent airway.

Recent advances in the treatment of obstructive sleep apnea have shifted the therapeutic paradigm from uniform airway-centered approaches toward phenotype-targeted and mechanism-informed interventions. This evolution reflects growing recognition that OSA arises from heterogeneous pathophysiological substrates, including anatomical compromise, impaired neuromuscular control, metabolic dysregulation, and altered central drive (Figure 3).

Among neuromodulatory strategies, hypoglossal nerve stimulation (HNS) represents a major translational breakthrough. By selectively activating the hypoglossal nerve in synchrony with inspiration, HNS enhances genioglossus muscle tone and restores upper airway patency during sleep. Beyond its mechanical effects, emerging evidence suggests that HNS may also engage central sensorimotor and autonomic circuits, potentially influencing brainstem–hypothalamic networks involved in respiratory–sympathetic coupling. Clinically, HNS demonstrates greatest efficacy in carefully selected patients with preserved neuromuscular responsiveness, highlighting the importance of phenotype-driven patient stratification.

In parallel, phenotype-targeted pharmacotherapies have begun to address non-anatomical contributors to OSA. Agents targeting metabolic dysfunction (e.g., GLP-1 receptor agonists) reduce upper airway collapsibility indirectly through weight loss and fat redistribution, while emerging pharmacological combinations aimed at enhancing upper airway muscle tone or central respiratory drive seek to correct neuromuscular deficits. Although these approaches differ in mechanism, their shared translational premise is that they may modulate upstream neural and metabolic contributors rather than passive airway stabilization. Critically, the expanding therapeutic landscape underscores that effective management of OSA will likely require mechanism-based matching of interventions to dominant pathophysiological traits, rather than reliance on a single modality. Integrating insights from PVN-centered autonomic and stress circuitry with neuromodulatory and pharmacological strategies may further refine patient selection and expand the spectrum of therapeutic benefit (44–46).

Importantly, several mechanistic aspects discussed in this review remain hypothetical. For instance, although oxidative stress and iron dysregulation are implicated in CIH models, there is currently no direct evidence demonstrating ferroptosis in PVN neurons in OSA. Similarly, proposed links between PVN mechanisms and therapeutic interventions such as positive airway pressure therapies, GLP-1 receptor agonists, AD109, or hypoglossal nerve stimulation remain speculative and require direct experimental validation.

## Conclusion

This review synthesizes current evidence suggesting that chronic intermittent hypoxia and chronic stress in OSA do not act independently but may converge on the paraventricular nucleus as an important integrative node. Within this framework, alterations in neuronal plasticity, neuroinflammation, oxidative stress, and epigenetic regulation may collectively contribute to sustained sympathetic activation and related cardiovascular and neuropsychiatric comorbidities. However, many of these mechanisms remain supported primarily by experimental models, and direct evidence in human OSA remains limited. Accordingly, the present review is best interpreted as a plausible integrative framework rather than a definitive mechanistic account.

### Future directions

To strengthen causal inference and improve biological resolution, future studies should directly characterize PVN neuronal phenotypes and cell death pathways in OSA-relevant models, with particular attention to whether oxidative stress and iron dysregulation converge on ferroptosis-related mechanisms in specific PVN cell populations. Given the cellular heterogeneity of the PVN, identifying subtype-specific vulnerability among CRH, oxytocinergic, GABAergic, and presympathetic neurons will be essential for clarifying how intermittent hypoxia and chronic stress reshape central autonomic and neuroendocrine regulation.

A second priority is the application of circuit-level approaches to dissect PVN connectivity within the broader autonomic–limbic network. Combining cell-type–resolved electrophysiology with optogenetic, chemogenetic, and projection-specific tracing strategies will be important for defining how the PVN interacts with the nucleus tractus solitarius, rostral ventrolateral medulla, and limbic structures under conditions of chronic intermittent hypoxia and systemic stress. Such studies will help move the field from correlational network models toward causal circuit-level explanations.

Future translational work should also determine whether proposed links between central PVN mechanisms and emerging therapeutic interventions can be experimentally validated. In particular, it remains important to clarify whether the benefits of phenotype-targeted strategies—such as positive airway pressure therapy, GLP-1 receptor agonists, AD109, and hypoglossal nerve stimulation—are mediated only through peripheral or mechanical pathways, or whether they also modulate central stress–autonomic circuitry relevant to PVN dysfunction (47).

In addition, circadian biology should be more explicitly integrated into experimental models of intermittent hypoxia and chronic stress. Because sleep fragmentation, circadian disruption, and altered temporal organization of neuroendocrine and autonomic function are likely to influence PVN network dynamics, incorporating these dimensions may yield a more realistic representation of the pathophysiological complexity of OSA and its comorbidities.

Finally, progress toward clinical translation will require the development of human-relevant biomarkers reflecting PVN-related dysfunction. Multimodal approaches integrating physiological phenotyping, nocturnal cardiovascular dynamics, neuroendocrine indicators, and neuroimaging-derived measures may help bridge mechanistic insights from experimental models with patient stratification, thereby supporting more precise identification of OSA subtypes and more individualized therapeutic decision-making.
